# Materia Medica

**Published:** 1866-02

**Authors:** 


### MATERIA MEDICA.

*Nux Vomica.* Gleanings from various authors.

*The Powder.*—The dose of powdered nux vomica usually prescribed is from three to five grains.

*The Extract.*—Of the alcoholic extract, the dose is half a grain.

*The Tincture.*—Until recently there has been but one tincture of nux vomica, and its dose is from five to ten minims. It is the old Dublin formula of 1826, (two ounces of nux vomica to eight ounces of rectified spirit,) which was adopted in the United States Pharmacopœia, and is the one given in Pareira and other works on materia medica.

The dose of the tincture of the new British Pharmacopœia, which is a much weaker preparation, is from half a drachm to a drachm.

It is usual to order nux vomica, in these doses, to be taken steadily three times a day for several weeks. But when a more powerful and speedy action is required, it is given in increasing doses cautiously, until some obvious effect is produced upon the system. Either mode might justly be considered as extremely safe, yet we have a case on record where it was otherwise.

Taylor, in his work on poisons, states that a lady taking three grains of powdered nux vomica three times a day, as directed, was compelled to discontinue its use on the sixteenth day, on account of the colic and purging it occasioned; five days afterwards, although not taking the remedy, she experienced ringing in the ears, drowsiness, increased sensibility to light and sound, and numbness and impairment of speech. And on the ninth day she lost her speech, and tetanic symptoms with twitchings of the muscles of the face and arms set in, as well as trismus. She swallowed with difficulty, her pupils became dilated, and her skin hot. On the twelfth day after discontinuing the nux vomica, she died exhausted from tetanic convulsions.

This exceptional case, occurring eight years since, has not seemed to affect the confidence of the profession in the remedy, for it is constantly prescribed in various diseases in a similar manner, and with impunity. And as the knowledge of its remedial powers is ever increasing, so is its employment becoming continually the more extended.

*Fatal Doses.*—Taylor speaks of two cases, in which fifty grains of powdered nux vomica proved fatal. And of another, where thirty grains of the powder in two doses of fifteen grains each, caused the death of a girl ten years of age. And, according to Guy, three grains of the alcoholic extract have likewise proved fatal.

It is unnecessary to dwell upon the excellent results obtained from nux vomica in pyrosis, gastrodynia, dysentery, colica pictonum, prolapsus of the rectum, tremor of drunkards, hysterical borborygmi, impotence, etc., etc., which are given in Pareira. We shall, therefore, take up the subject where it is there left off, and quoting from Stille's admirable work on materia medica, and from other sources, endeavor to give our readers an additional and more recent synopsis of the opinions of the profession concerning this agent.

*Paraplegia.*—Dr. Brown Sequard says that nux vomica should be avoided as a most dangerous poison, in all cases of paraplegia in which there are signs of congestion or inflammation of the spinal cord or its meninges, for in these it but increases the cause of the paralysis, and produces an aggravation of the symptoms. He says there are two distinct groups of cases of paraplegia, one distinguished by symptoms of irritation, the other characterized by the absence of them. The symptoms of irritation observed in the former class, are convulsions, cramps, twitchings, erection of the penis, formication, and itching; diminution of temperature, wasting of the muscles, œdema, bed sores, and alkaline urine. In the second class all these symptoms are wanting, and the paraplegia is caused by the white or non-inflammatory softening, or is of the reflex kind; for this class nux vomica is particularly applicable, from the power it possesses of augmenting the amount of blood sent to the spinal cord and membranes, and, from the extra nutrition thereby derived, of increasing the vital properties of this nervous centre. *Braithwaite* $\frac{43}{26}$.

*Recurring Hordeolum.*—Dr. S. C. Sewell of Ottawa, states that he has lately been very successful in the treatment of obstinate stye by means of small continuous doses of tincture of nux vomica, and gives two cases in illustration. The first, that of a young lady whose eyes for upwards of four years had never been entirely free from styes, and who had lost her eyelashes from them. He prescribed four minim doses of the Dublin tincture twice a day, and found the effect immediate; for the stye then forming, receded, and she has not been troubled with more than two or three of them since, (now two years and a half.) She took the medicine regularly for six weeks, and has twice resorted to it for a similar period since. The other was that of a girl of fifteen, who for two years had constantly been troubled with styes, and this too was cured by four minim doses of tincture of nux vomica. The stye she had on her eye at the time, suppurated, but she never had another one afterwards (now two years.) He says that cases of recent hordeolum yield quite as readily to this treatment as those of long standing.

*Abscess of the Labia Pudendi.*—Dr. Sewell also relates a case of obstinate recurring abscess of the labia, which regularly made its appearance a day or two before or after the menstrual period. The lady had thus been afflicted nearly every month for four or five years, and had consulted a great number of physicians in vain for relief. On inquiry he found that they first made their appearance on the cessation of obstinate styes, with which she had previously been troubled for a couple of years. Considering the case vicarious to the styes, he put her at once upon six minim doses of tincture of nux vomica, giving it twice a day as before. The treatment proved immediately successful, and up to the present (now a year and a-half) she has not been troubled with them but once or twice.

*In Skin Diseases.*—Dr. Sewell remarks that he has found nux vomica to produce an excellent effect on skin diseases occurring in cachetic or scrofulous subjects, by rendering them more readily amenable to local treatment, and instances impetigo of the scalp in particular. For a similar reason he also suggests its employment in strumous ophthalmia.

*In Tetanus*—In 1847, Dr. Fell of New York, published seven cases of tetanus, six of which were certainly of the traumatic variety, and which all recovered under its use. His plan of administering it was to give an eighth or a tenth of a grain of strychnia, and in two hours a sixteenth of a grain, thus reducing the dose still further, and only to the extent of producing specific signs of its influence after each one. Dr. Kolloch, also relates a case of traumatic tetanus, occurring in a negro girl, which was cured by strychnia, given in doses of a twelfth of a grain every two hours. *Stille* $\frac{2}{245}$.

*In Hysterical Spasm of the Œsophagus.*—We have the report of a case which yielded to increasing doses of nux vomica, continued until the system became affected.

*In Prolapsus of the Rectum.*—Koch of Stuttgart, speaks of a cure he effected in a case of fifteen years standing, by the employment of cold water injections, medicated by the addition of twelve drops of tincture of nux vomica. And Dr. A. Johnson has been equally successful by the application of strychnia (a sixteenth of a grain) to a blistered surface over the coccyx. *Oper. cit.*

*In either Incontinence or Retention of Urine.*—When depending on impaired power in the muscular coat of the bladder from habitual distension, or from pressure by the uterus, the operation of nux vomica is generally very efficient. It has been employed in cases occurring after parturition. And Solly has given it successfully in incontinence after lithotomy. Lecluyse, in retention from paralysis, injected a solution of strychnia into the bladder. In incontinence of urine in children, Mondiere, Ribes, Guersent, Mauricet and others, found the alcoholic extract of nux vomica better than all other remedies. Trousseau, however, thinks belladonna superior. *Oper cit.*, $^{2}_{245}$.

*In the Vomiting of Pregnancy.*—Dr. Kroyher of Presburg, considers the tincture of nux vomica a specific. He directs a few drops to be taken in a little aromatic or cherry-laurel water, increasing it to ten, twelve, or eighteen drops, if necessary, every morning early, and in the evening. Br. $^{3}_{156}$.

*In Hay Fever.*—Mr. Gream has found it very useful in removing the coryza. He orders from ten to twenty drops of the old tincture three times a day, and the application of Goulard's cerate to the nose. *Braithwaite* $^{22}_{7}$.

*In Facial Neuralgia.*—Dr. Rœlants of Rotterdam, has furnished a favorable account of the treatment of both old and recent cases of facial neuralgia by nux vomica. Twenty-five out of twenty-nine, he states, were cured and three were still under treatment. All he says, yielded to the remedy with singular rapidity. *Stille* $^{2}_{247}$.

*In Lead Colic.*—Nux vomica is highly recommended by Dr. Serres, Dr. Huss of Stockholm, Dr. Neligan of Dublin, and Drs. Swett and Bulkley of New York. The dose of the Dublin tincture is from ten to thirty drops according to the course of the disease; it is to be administered also in clysters, and applied to the abdomen on cataplasms. It generally gives relief in forty-eight hours, the bowels acting and the pain subsiding.

*In Chronic Rheumatism.*—The external use of equal parts of tincture of nux vomica and soap liniment is strongly recommended by Kessel. *Stille* $^{2}_{247}$.

*In Dysmenorrhœa.*—Rademacher combines tincture of nux vomica with tincture of castor in equal proportions, and directs thirty drops to be taken five or six times a day.

*In prolonged after-pains.*—He finds it to give relief when administered in a similar manner.

*Gastric Irritability.*—There are various forms in which this remedy may prove extremely beneficial. In true gastralgia, a disease in which paroxysmal pains of various characters, but always intensely severe, are felt in the stomach and radiates from thence to the chest, hypochondria, and back, followed by the eructation of gas and insipid or acid liquid. Rowland gives a quarter of a grain of the extract of n. v. in such cases, three or four times a day.

In irritability accompanied by gnawing pain at the pit of the stomach and vomiting of food, Dr. Huss prescribes one grain of powdered nux vomica with ten grs. of magnesia, three times a day, increasing every third dose by half a grain. He says that it often gives instant relief, and does not require continuance longer than from ten days to a fortnight.

In cases of gastric irritability in which the patient is anæmic, and iron cannot be borne, small doses of nux vomica enables the system to tolerate and derive benefit from ferruginous remedies.

*In Dyspepsia.*—Werber has found it of signal benefit when the biliary secretion is defective, the digestion slow, the appetite impaired, the bowels torpid, and the spirits depressed. A condition which is apt to follow excesses in study or business, in eating, drinking alcohol liquors, tea or coffee, and in venereal indulgence. *Stille* $\frac{2}{248}$.

*In Constipation.*—Drs. Copeland, Neligan, Clark and others, recommend nux vomica in all cases depending merely on deficient tone of the muscular coat of the bowels, and an imperfect propelling power in the upper part of the rectum, *Braithwaite* $\frac{33}{158}$. All alike agree that it should be combined with some gentle purgative to promote its action. Mr. Boult of Bath, finds a pill of half a grain of the alcoholic extract of nux vomica, $\frac{3}{4}$ of a grain of aloes, and as much rhubarb to act nicely in such cases, and never to lose its laxative power although taken daily for months. He says that he has never derived much benefit from nux vomica alone in costiveness. Br. $\frac{18}{133}$. An excellent resume of the opinions of the profession on the effects of this remedy in costiveness may be found in Braithwaite $\frac{34}{127}$. Dr. Byford in his new work, reviewed in this issue, also adds his testimony to the usefulness of nux vomica for the removal of constipation. And although advising watchfulness during its employment, states that he has never noticed any evil effect from its use, beyond a slight inconvenience in the way of nervous startings, although constantly administered for weeks [134]. A favorite prescription of his is five grains of powdered nux vomica with a grain of quinine three times a day after meals; but he often orders it likewise with iron by-hydrogen in a similar manner [132].

*In Spasmodic Obstruction of the Bowels.*—Vidal procured relief by using a sixteenth of a grain of strychnia every four hours. Dr. Parker of Charleston, has likewise reported a case of obstruction of the bowels, which, after resisting various purgatives and enemata, yielded to strychnia, given in doses of a twelfth of a grain three times a day. Homolle is stated not only to have removed impacted fæces by its means, but actually to have relieved strangulated hernia, when the necessity of an operation seemed to be inevitable. Stille $\frac{2}{249}$.

*Dysentery.*—The tonic influence of this remedy upon the bowels is farther shown by its efficacy in some forms of dysentery. In the last century, Hagstrom employed powdered nux vomica in scruple doses with wonderful success. Hufeland also resorted to it, with the happiest results, in an epidemic of dysentery at Jena, in 1795. He prescribed rather less than a grain of the extract every two hours. In his Enchiridion (p. 366,) he directs but ten grains of the powder daily, and this only after other means have failed. Rademacher has found it occasionally necessary to combine it with opium. Mr. Vaux of Ipswich, gave as much as seven grains of the powder three times a day, and reported his success as remarkably uniform. Frisch prescribed it with advantage in sub-acute dysentery, and Ricamier in chronic diarrhœa.

*Diarrhœa from Exhaustion.*—Dr. Nevins of Liverpool, highly recommends the employment of nux vomica in diarrhœa from exhaustion, and especially when occurring among the poor and in children. He was led to its adoption from the frequent disappointment he experienced in the employment of astringents and ordinary tonics in such cases. His favorite prescription is as follows:

Alcoholic extract nux vomica, pulv. rhubarb, and blue-pill, of each half-a-grain; saccharine carbonate of iron, one grain; opium, an eighth of a grain. M. To be made into one pill if for an adult, or more if for children.
S. One such pill to be taken three times a day.

In many cases he omits the opium entirely. He says that nux vomica exalts the nervous energy of the bowels, and enables the lacteals to absorb the nutriment from the food, whilst the iron is allowed to act as a tonic, and the rhubarb and blue pill to improve the secretions. A change for the better is generally perceptible in a few days, and he has seldom occasion to continue the prescription longer than a fortnight. Braith. $\frac{19}{105}$.

Dr. Bardsley has published six cases of chronic diarrhœa in persons advanced in life, and of feeble constitution, which were cured by the extract of nux vomica, administered in doses of a sixth of a grain three times a day. Stille $\frac{2}{250}$. W. E. B.
—*Canada Lancet.*

